# Differentiating Botulinum Neurotoxin-Producing Clostridia with a Simple, Multiplex PCR Assay

**DOI:** 10.1128/AEM.00806-17

**Published:** 2017-08-31

**Authors:** Charles H. D. Williamson, Adam J. Vazquez, Karen Hill, Theresa J. Smith, Roxanne Nottingham, Nathan E. Stone, Colin J. Sobek, Jill H. Cocking, Rafael A. Fernández, Patricia A. Caballero, Owen P. Leiser, Paul Keim, Jason W. Sahl

**Affiliations:** a Pathogen and Microbiome Institute, Northern Arizona University, Flagstaff, Arizona, USA; b Bioscience Division, Los Alamos National Laboratory, Los Alamos, New Mexico, USA; c Molecular and Translational Sciences Division, United States Army Medical Research Institute of Infectious Diseases, Frederick, Maryland, USA; d Informatics, Computing and Cyber Systems, Northern Arizona University, Flagstaff, Arizona, USA; e Área Microbiología, Departamento de Patología, Facultad de Ciencias Médicas, Universidad Nacional de Cuyo, Centro Universitario, Mendoza, Argentina; University of Helsinki

**Keywords:** Clostridium botulinum, whole-genome sequencing, PCR, biomarker

## Abstract

Diverse members of the genus Clostridium produce botulinum neurotoxins (BoNTs), which cause a flaccid paralysis known as botulism. While multiple species of clostridia produce BoNTs, the majority of human botulism cases have been attributed to Clostridium botulinum groups I and II. Recent comparative genomic studies have demonstrated the genomic diversity within these BoNT-producing species. This report introduces a multiplex PCR assay for differentiating members of C. botulinum group I, C. sporogenes, and two major subgroups within C. botulinum group II. Coding region sequences unique to each of the four species/subgroups were identified by *in silico* analyses of thousands of genome assemblies, and PCR primers were designed to amplify each marker. The resulting multiplex PCR assay correctly assigned 41 tested isolates to the appropriate species or subgroup. A separate PCR assay to determine the presence of the *ntnh* gene (a gene associated with the botulinum neurotoxin gene cluster) was developed and validated. The *ntnh* gene PCR assay provides information about the presence or absence of the botulinum neurotoxin gene cluster and the type of gene cluster present (*ha* positive [*ha*^+^] or *orfX*^+^). The increased availability of whole-genome sequence data and comparative genomic tools enabled the design of these assays, which provide valuable information for characterizing BoNT-producing clostridia. The PCR assays are rapid, inexpensive tests that can be applied to a variety of sample types to assign isolates to species/subgroups and to detect clostridia with botulinum neurotoxin gene (*bont*) clusters.

**IMPORTANCE** Diverse clostridia produce the botulinum neurotoxin, one of the most potent known neurotoxins. In this study, a multiplex PCR assay was developed to differentiate clostridia that are most commonly isolated in connection with human botulism cases: C. botulinum group I, C. sporogenes, and two major subgroups within C. botulinum group II. Since BoNT-producing and nontoxigenic isolates can be found in each species, a PCR assay to determine the presence of the *ntnh* gene, which is a universally present component of *bont* gene clusters, and to provide information about the type (*ha*^+^ or *orfX*^+^) of *bont* gene cluster present in a sample was also developed. The PCR assays provide simple, rapid, and inexpensive tools for screening uncharacterized isolates from clinical or environmental samples. The information provided by these assays can inform epidemiological studies, aid with identifying mixtures of isolates and unknown isolates in culture collections, and confirm the presence of bacteria of interest.

## INTRODUCTION

Diverse members of the genus Clostridium produce botulinum neurotoxins (BoNT), which block nerve function, causing paralysis. BoNT-producing clostridia have been separated into groups based upon physiological attributes such as optimal growth temperature, proteolytic differences, and differential fermentation of sugars, and a variety of genetic and genomic analyses also demonstrated that the groups are separate species (see references 1, 2, and 3 and references therein). Currently, known species or groups that include BoNT-producing isolates are Clostridium botulinum group I, C. botulinum group II, C. botulinum group III, C. argentinense, C. baratii, C. butyricum, and C. sporogenes. These species have different physiological attributes that are useful for differentiation and have an impact on their ecology and disease presentation. For example, C. botulinum group II organisms have lower optimal growth temperatures than other species and can grow and produce toxin at near-freezing temperatures (4–6), which indicates that group II organisms may pose a risk for the occurrence of foodborne botulism cases originating from consumption of vacuum-packed, refrigerated foods (7, 8). Cold tolerance of group II members may also be a factor in the prevalence of botulism caused by BoNT type E (BoNT/E) (produced by some C. botulinum group II members) in colder regions.

Botulinum neurotoxins have been classified into seven toxin types that are serologically distinct (BoNT/A to BoNT/G), all of which have been associated with botulism intoxications except BoNT/G. Some organisms produce multiple toxin types, and some toxin types can be expressed by bacteria representing multiple species. For example, C. botulinum group I organisms produce toxin types A, B, and/or F whereas group II organisms produce toxin type B, type E, or type F (1, 2, 9–11). Recent whole-genome sequencing analyses have also placed BoNT/B-producing isolates within C. sporogenes (12, 13). Human botulism cases have been most commonly attributed to members of groups I and II (7, 14, 15).

Molecular techniques and whole-genome comparisons have been applied to study the diversity of BoNT-producing clostridia. As C. botulinum group I and group II members are commonly isolated from environmental and clinical samples, isolates within these two species have been the focus of many studies. Recently, comparative genomics studies differentiated C. sporogenes and members of C. botulinum group I (12, 13, 16–18). Both C. sporogenes strains and members of C. botulinum group I may produce botulinum neurotoxins or be nontoxic (12, 13). The diversity of C. botulinum group II members has also been evaluated with comparative genomics techniques, with results indicating that group II contains significant diversity at the genomic level and can be divided into two major subgroups (13, 18–20). C. botulinum group I, C. sporogenes, and the two major subgroups of C. botulinum group II are the focus of this study.

Initial confirmation of botulism involves detection and identification of the BoNTs that might be present. Historically, this was done by analyzing sample material or enrichment cultures (anaerobic broth cultures that promote the growth of organisms of interest) using mouse bioassays (21). This technique is being replaced by rapid mass spectrometry assays that measure BoNT enzymatic activity (22) or PCR-based assays that detect the presence of *bont* genes (genes associated with the botulinum neurotoxin) (23–34). While knowledge of the toxin type is important for clinical reasons, it does not provide information about the phylogenetic placement of the isolate within BoNT-producing species. Information about the phylogenetic placement of an isolate can be useful for epidemiology studies and for screening culture collections or enrichment cultures.

Multiple researchers have developed methods for differentiating BoNT-producing clostridia beyond toxin type. Flagellin gene diversity has been used for typing C. botulinum group I and II isolates (35, 36). In a 2008 study, Dahlsten and colleagues developed a PCR assay for differentiating C. sporogenes and group I isolates from group II isolates based on the presence of the *fldAIBC* gene cluster (37). Weigand and colleagues (12) presented a number of markers for identifying members of C. sporogenes. The goal of this study was to build on previous works and to develop a PCR assay capable of easily identifying members of four BoNT-producing species/subgroups: C. botulinum group I, C. sporogenes, and two major subgroups of C. botulinum group II. The use of a phylogenetic marker paired with the knowledge of the toxin type(s) provides a useful tool for characterizing BoNT-producing isolates.

## RESULTS

### Differentiation of isolates with *rpoB* and core genome SNP phylogenies.

**FIG 1:**
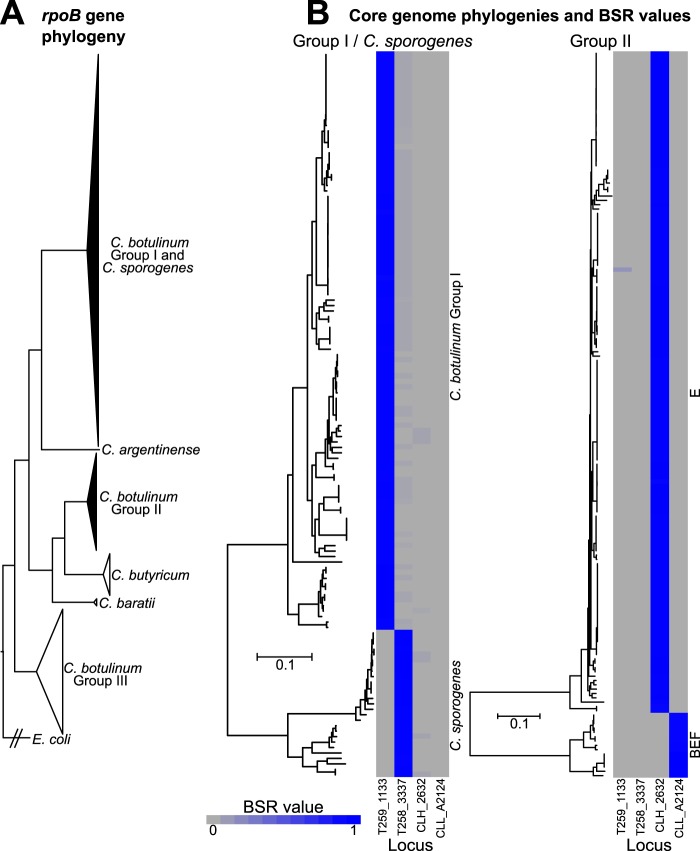
Phylogenies differentiating BoNT-producing bacteria and heat maps of BSR values. (A) BoNT-producing clostridia were differentiated with a phylogeny inferred using an alignment of *rpoB* genes. (B) Members of C. botulinum group I, C. sporogenes, and members of C. botulinum group II were further delineated with core genome SNP phylogenies. The SNP phylogenies differentiate C. botulinum group I from C. sporogenes and separate C. botulinum group II into two major subgroups. The heat maps alongside the core genome SNP phylogenetic trees indicate the BSR values of the markers identified with LS-BSR (76). BSR values are used as a measure of the relatedness of coding region sequences between genomes. Blue boxes indicate the presence of the coding region sequence in a genome assembly, while gray boxes indicate that the coding region sequence is absent.

BoNT-producing clostridial genomes were analyzed for markers that could be used for the development of PCR-based assays to differentiate between closely related species. Isolates representing various BoNT-producing clostridial species were differentiated with phylogenies inferred using alignments of the *rpoB* gene. Core genome single nucleotide polymorphism (SNP) phylogenies were used to further differentiate isolates within C. botulinum group I and C. sporogenes as well as C. botulinum group II (Fig. 1; Table 1; see also Data Set S1 in the supplemental material). C. botulinum group I and C. sporogenes isolates (*n* = 133) were differentiated by the use of a core genome SNP phylogeny (Fig. 1) (retention index = 0.92) inferred using a 171,364-character SNP matrix generated from a 1,504,184-character core genome alignment. BoNT-producing and nontoxic isolates are present in both C. botulinum group I and C. sporogenes. These results are consistent with recent evaluations of C. botulinum group I-C. sporogenes diversity (12, 13, 16–18). Currently available whole-genome sequence data indicate that, while the botulinum neurotoxin gene cluster is present within the chromosome and on plasmids within C. botulinum group I, toxin gene clusters in C. sporogenes appear to be present only on plasmids.

**TABLE 1:** Isolates subjected to whole-genome sequencing in this study

BioSample accession no.	GenBank accession no.	Isolate identifier^*a*^	Collection date	Collection location	Source	BoNT^*b*^
SAMN06022741	MWJA00000000	Clostridium botulinum group I BrDuraAf	1987	Argentina	Outbreak	Af
SAMN06022746	MWJE00000000	Clostridium botulinum group I CDC1656	1977	USA: Utah	Infant	B
SAMN06022740	MWIZ00000000	Clostridium botulinum group I Man216	1987	Argentina	Herb	F
SAMN06022747	MWJF00000000	Clostridium botulinum group I Prevot 1542	1956		Ham	B
SAMN06022737	MWIW00000000	Clostridium botulinum group I SU0305	1987	Argentina	Soil	B
SAMN06022739	MWIY00000000	Clostridium botulinum group I SU0632	1987	Argentina	Soil	F
SAMN06022736	MWIV00000000	Clostridium botulinum group I SU0972		Argentina	Soil	A
SAMN06022749	MWJH00000000	Clostridium botulinum group I SU1033	1998	Argentina	Soil	NT
SAMN06022738	MWIX00000000	Clostridium botulinum group I SU1297	1987	Argentina	Soil	B
SAMN06022742	MWJB00000000	Clostridium botulinum group II Bac-01-03998		USA: New York	Bald eagle (stomach contents)	E
SAMN06022743	MWJC00000000	Clostridium botulinum group II Bac-02-06430		USA: New York	Round goby	E
SAMN06022744	SRR5168317	Clostridium botulinum group II Bac-03-06093		USA: New York	Herring gull	E
SAMN06022745	MWJD00000000	Clostridium botulinum group II Bac-04-16057		USA: New York	Common loon	NT
SAMN06022748	MWJG00000000	Clostridium botulinum group II Prevot Ped 4				E
SAMN06022751	MWJJ00000000	Clostridium sporogenes CLS_DGF_0088_06	2014	USA: Flagstaff, AZ	Canine fecal sample	NT
SAMN06022750	MWJI00000000	Paraclostridium bifermentans SU1074NT	1998	Argentina	Soil	NT

a The Bac-01-03998, Bac-02-06430, Bac-03-06093, and Bac-04-16057 strains originated at the Wadsworth Center, New York State Department of Health, Albany, NY (62).

b NT, no-botulinum neurotoxin.

C. botulinum group II isolates (*n* = 168) were divided into two major subgroups by a core genome SNP phylogeny (Fig. 1) (retention index = 0.95) inferred using a 135,135-character matrix generated from a 1,969,738-character core genome alignment. One subgroup includes members that produce BoNT/E, labeled the “E” subgroup here. The second is labeled the “BEF” subgroup here. The BEF subgroup consists of multiple BoNT/B4 and BoNT/F6 strains, plus the unusual BoNT/E9 strain that was located in Argentina (19). Three members of this subgroup included in this study are nontoxic strains, two of which were originally reported to produce BoNT/E toxin (20). Recent studies demonstrated similar results in evaluating group II isolates using whole-genome sequence data (18, 20), and average nucleotide identity (ANI) values have also been used to demonstrate the diversity of C. botulinum group II isolates (13, 19). Stringer and colleagues (38) have identified genetic and physiological differences, specifically, carbohydrate fermentation differences, between members of the E and BEF subgroups.

### Marker identification.

Genome assemblies representing 458 clostridial isolates (Data Set S1) were evaluated with the large-scale BLAST score ratio (LS-BSR) tool (BLAT alignment option) to identify coding region sequences unique only to targeted species or subgroups of BoNT-producing bacteria. The targeted species/subgroups included C. botulinum group I, C. sporogenes, the C. botulinum group II E subgroup, and the C. botulinum group II BEF subgroup. Coding region sequences present in all target genomes (those present in all genomes with BSR values of ≥0.8) were queried against all other clostridium genomes outside the targeted species/subgroup. PCR primers were designed to amplify target coding region sequences that displayed very low BSR values in other genomes. BSR values are used as a measure of the relatedness of a given coding region sequence between two genomes (39). Additionally, PCR primers were screened against nontarget genomes to minimize unintended priming sites. A single marker was selected for each species or subgroup (see Table 2). The average BSR value of markers within the intended target genomes was above 0.96 (minimum BSR value = 0.92), while the average BSR value of markers outside the intended target genomes was below 0.03 (maximum BSR value = 0.36) (see Table 2; see also Data Set S1).

The four markers were also screened against 5,402 publicly available genome assemblies representing bacteria outside the clostridia (Data Set S2). The maximum BSR value of the identified markers in the screened nonclostridium genomes was 0.23. The BSR values indicate that the markers are highly conserved within the targeted genomes and appear to be absent in genome assemblies representing bacteria outside the targeted species/subgroups. Thus, PCR amplification of the markers should provide insight into the phylogenetic placement of the analyzed isolate.

### Multiplex PCR assay development.

**FIG 2:**
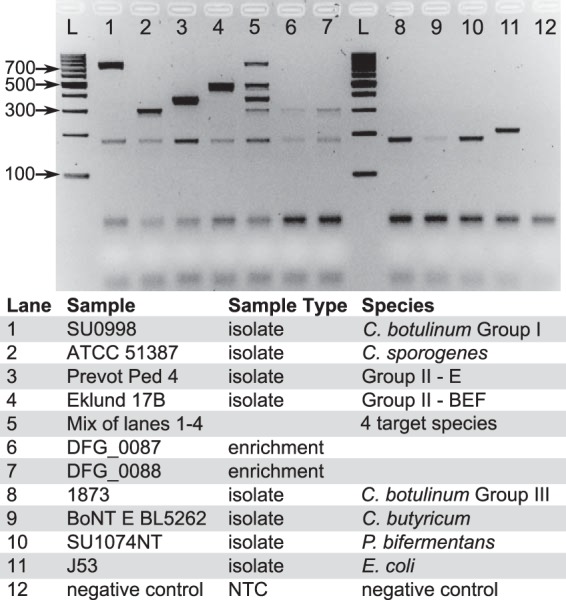
Assessment of multiplex marker PCR amplicons with gel electrophoresis. Target genome amplicons have the following approximate sizes: C. botulinum group I, 775 bp; C. sporogenes, 310 bp; C. botulinum group II E subgroup, 375 bp; C. botulinum group II BEF subgroup, 480 bp. An internal amplification control targets the 16S rRNA gene, producing an amplicon of approximately 180 bp. Two bands indicate a positive reaction for one of the targeted species or subgroups (lanes 1 to 4). Lane 5 illustrates a mixture of PCR products of all four targeted species/subgroups. Lanes 6 and 7 illustrate the ability of the assay to identify target species/subgroups (in this case, C. sporogenes) in a mixed culture of unknown clostridia. Lanes 8 to 11 include isolates of nontarget taxa, and lane 12 includes a negative control. The numbers to the left of the gel indicate the size (in base pairs) of the ladder bands. Sample bands smaller than 100 bp likely represent primer dimers. NTC, no-template control.

A multiplex PCR assay was developed to amplify markers for identifying members of C. botulinum group I, C. sporogenes, the C. botulinum group II E subgroup, and the C. botulinum group II BEF subgroup. Primers specific for each marker and for the 16S rRNA gene, which was used as an internal amplification control, were included in each reaction (see Table 3). The PCR products for target-specific primer pairs and the 16S rRNA gene range in size from approximately 180 to approximately 775 bp. The size of the band on an agarose gel corresponds to the marker designed for each targeted species or subgroup, allowing easy visualization of positive reactions (see Table 3) (Fig. 2). The multiplex PCR and subsequent gel electrophoresis can easily be performed in a matter of hours, providing a quick tool for characterizing commonly encountered BoNT-producing bacteria.

DNA extractions representing 41 different bacterial isolates were screened with the multiplex PCR assay (see Table S1 and Fig. S1 in the supplemental material). The phylogenetic relationships of these isolates were determined with core genome SNP phylogenies (Fig. 1). Of the 37 isolates that are members of the targeted species/subgroups, all 37 were correctly assigned to the appropriate species or subgroup by the PCR assay. None of the four nontarget isolates were erroneously identified as members of any targeted species or subgroup. The results of screening representative isolates are presented in Fig. 2.

Detection of BoNTs and *bont* genes, particularly in infant botulism, is often accomplished using fecal samples. The use of these samples can be challenging due to the presence of interfering substances and material from an array of bacterial species. Therefore, sample materials are often incubated anaerobically in broth cultures to enrich for clostridia. To test the efficacy of the multiplex PCR assay for identifying the targeted clostridia in mixed bacterial cultures, two enrichment cultures obtained from domesticated-canine fecal samples were screened (40). These samples were originally used in a study investigating the role of pet dogs in C. difficile infections in humans (40). The multiplex PCR results indicated that at least one member of C. sporogenes was present in both fecal enrichments. Whole-genome sequencing of an isolate from one of the fecal enrichments (DGF_0088) identified the isolate as a non-BoNT-producing member of C. sporogenes (CLS_DGF_0088_06; BioSample SAMN06022751), which is consistent with the results of the multiplex PCR assay. As the use of enrichment cultures is a common practice in attempting to detect or isolate BoNT-producing clostridia from clinical or environmental samples, the multiplex assay can be a useful tool for screening these mixed cultures for members of the targeted species or subgroups.

The sensitivity of the multiplex PCR assay in a complex DNA sample was estimated by mixing DNA extracted from a C. botulinum group I isolate (SAMN06022737; BoNT/B SU0305) into DNA extracted from a canine fecal sample and screening the resulting DNA mixtures. Though a very faint band was produced on a 2% agarose gel, the assay successfully identified the C. botulinum group I isolate DNA when ∼2,400 16S rRNA gene copies (∼270 genome equivalents) were present in the reaction (Fig. S2). While the assay sensitivity is appropriate for its purpose of screening isolate and enrichment cultures, it may be improved by adjusting the number of PCR cycles or loading a larger reaction volume on a gel.

### *ntnh* gene PCR assay.

The nontoxic nonhemagglutinin gene (*ntnh*) is present upstream of the botulinum neurotoxin gene in all known botulinum neurotoxin gene clusters (9, 41). Thus, detection of this gene by PCR indicates that a screened isolate or enrichment culture contains at least one *bont* gene cluster. While this method cannot differentiate between *bont* clusters with active toxin genes and those with silent toxin genes (23, 24), to date, no clostridial isolates have been identified as having a silent toxin gene with no accompanying active *bont* gene cluster [e.g., within BoNT/A1(B)-producing organisms]. Primers were developed to amplify the *ntnh* gene, and primer coverage was evaluated *in silico* with PrimerProspector by querying the primers against near-full-length *ntnh* genes extracted from genome assemblies. Of the 138 near-full-length sequences analyzed, PrimerProspector predicted PCR amplification of 137 (Table S2). Amplicons of two sizes were predicted; *ntnh* genes associated with *orfX*^+^ (presence of three open reading frames, *orfX1*, *orfX2*, and *orfX3*) toxin gene clusters generated amplicons that were approximately 225 bp in size, while *ntnh* genes associated with *ha*^+^ (presence of three genes encoding the hemagglutinin proteins HA-70, HA-33, and HA-17) toxin gene clusters generated approximately 325-bp amplicons (Table S2). The differently sized bands are a result of the primers targeting regions on either side of a portion of the *ntnh* gene encoding the nLoop (Fig. S3), which has been shown to be absent from nontoxic nonhemagglutinin proteins associated with some BoNT/A and all BoNT/E and BoNT/F serotypes and present in nontoxic nonhemagglutinin proteins associated with some BoNT/A and all BoNT/B, BoNT/C, BoNT/D, and BoNT/G serotypes (42). Thus, the PCR assay is also capable of providing information regarding the *bont* gene cluster type (*ha*^+^ or *orfX*^+^).

DNAs representing 41 bacterial isolates were screened with the *ntnh* gene PCR assay (Table S1; Fig. S4). Bands of appropriate size indicated positive reactions for the *ntnh* gene PCR assay. Positive reactions in the *ntnh* PCR assay were identified for all isolates in which *bont* genes were identified in the genome assembly. *ntnh* bands of both sizes are visible in lane 19 (Fig. S4), as this strain is a BoNT/A1(B) strain [A1, *orfX*^+^; (B), *ha*^+^]. To ensure that the primers targeted the *ntnh* gene, the amplicon from DNA representing Biosample SAMN03779976 (BoNT/A2 SU0998) was sequenced. BLAST results and alignment (MUSCLE aligner; Fig. S3) of the amplicon sequence to reference *ntnh* gene sequences indicated that the primers targeted the *ntnh* gene.

### Evaluation of published markers.

Dahlsten and colleagues developed a PCR assay targeting the *fldAIBC* gene cluster (37). As those authors noted, the assay targets a gene present in a number of bacteria and is designed to differentiate BoNT-producing isolates previously identified as group I or II (37). Weigand and colleagues (12) identified markers specific to C. sporogenes. LS-BSR analyses indicated that both approaches performed well for the genomes tested here. Our marker approach is designed to identify members of four targeted species/subgroups without prior knowledge of serotype or phylogenetic placement.

## DISCUSSION

Botulinum neurotoxins, produced by diverse clostridia, cause a severe form of paralysis known as botulism. BoNT-producing clostridia have been cultivated from many sources (43–51), and biochemical attributes and BoNT typing are used to characterize isolates (23). Both gel-based and quantitative PCR (qPCR) assays have been developed to identify the BoNT serotype of an isolate (24–34). However, these assays may not provide information regarding the phylogenetic placement of the isolate in question. For example, BoNT/B-containing isolates can be found in three different categories of clostridial species: C. botulinum group I, C. sporogenes, and C. botulinum group II. Therefore, identifying the presence of the *bont*/B gene does not assist in phylogenetic analysis of the isolate. Additionally, a nontoxigenic isolate cannot be detected with assays targeting *bont* gene clusters, though these bacteria may still be of interest. Such isolates may be nontoxic members of these species, or they may be isolates that were originally toxic but lost toxicity over time, an occurrence which is particularly prevalent with organisms where the *bont* genes are found within plasmids, as these plasmids can be easily lost on repeated culture. While phylogenetic relationships can be determined by methods such as multilocus sequence type (MLST) gene sequencing (52, 53) or whole-genome analyses (12, 13, 16, 17, 20), these methods do not provide a rapid, inexpensive assay that can be performed in most laboratories. The multiplex marker PCR assay presented here can quickly differentiate isolates within C. botulinum group I, C. sporogenes, and two major subgroups of C. botulinum group II. The assay can also be used to identify these bacteria in mixed cultures or potentially in environmental or clinical samples, which may be challenging using alternative assays.

Schill and colleagues (54) evaluated a number of isolates that were all labeled as representative of C. sporogenes PA 3679. The authors found that the isolates formed two genetically distinct clades. The PA 3679 isolates have also been shown to have variable heat resistance, which should be considered carefully for food safety evaluations (55). Consistent with these findings, our analyses indicated that some of the isolates labeled as representing PA 3679 belong to C. botulinum group I (SAMN03418480, SAMN03418522, SAMN03418523, SAMN03418527, and SAMN03418524), while other isolates labeled as representing PA 3679 fall within C. sporogenes (SAMN03418528, SAMN03418526, and SAMN03418525). *In silico* analyses indicated that the multiplex marker PCR assay presented here can differentiate between these two different groups of isolates labeled with the same strain identifier. Situations such as that described above highlight the utility of this assay.

The presence or absence of a botulinum neurotoxin gene cluster within a bacterial culture can be determined by detecting the presence of the *ntnh* gene using PCR and gel electrophoresis. The *ntnh* gene PCR assay described here also provides information about the type of toxin gene cluster (*ha*^+^ or *orfX*^+^) present in the DNA being tested. By coupling the *ntnh* gene PCR assay and the multiplexed marker PCR assay, an isolate can be putatively designated a BoNT-producing isolate and placed into a phylogenetic context. The results of these assays can be confirmed with PCR assays designed to identify BoNT serotypes or markers specific to one species (e.g., the markers for C. sporogenes designed by Weigand et al. [12]). As the genomes of more BoNT-producing isolates are sequenced, the marker assay can be further tested and updated if necessary. The information provided by the assays can be used to inform epidemiological studies, confirm the identity of isolates before whole-genome sequencing, or screen mixed cultures for bacteria of interest.

## MATERIALS AND METHODS

BoNT-producing clostridia within C. botulinum group I, C. sporogenes, and C. botulinum group II were differentiated using core genome SNP phylogenies. Markers for detecting four species/subgroups of BoNT-producing clostridia were identified by analyzing whole-genome sequence data with BLAST score ratio analyses, and a multiplex PCR assay was developed for rapid detection of these species/subgroups. Additionally, PCR primers targeting the *ntnh* gene were developed for detecting the presence of *bont* gene clusters.

### Acquisition of genomic sequence data.

Genomic DNA or isolates were provided by collaborators. For the isolates, DNA was extracted with DNeasy kits (Qiagen, Valencia, CA, USA). Whole-genome sequencing was conducted on the Illumina platform (GAIIx and MiSeq) at TGen North (Flagstaff, AZ). Genomes were assembled with UGAP (https://github.com/jasonsahl/UGAP), using Trimmomatic (56) for adapter trimming, BayesHammer (57) for read error correction, the SPAdes assembler (58) for contig assembly, and Pilon for genome polishing (59). Additionally, due to the number of contigs and ambiguous characters in the original assembly, Image (60) was used to improve the assembly for SAMN06022746. Contaminating contigs were identified with BLAST (61) searches of the first 200 nucleotides of each contig against the NCBI nonredundant nucleotide database, and the results were confirmed with BLAST searches of the entire contig. Contigs associated with contamination or with anomalously low coverage were manually removed from assemblies. Sequenced isolates are listed in Table 1.

Publicly available genome assemblies and read data (Data Sets S1 and S2) were downloaded from NCBI. Multiple assemblies (or read data) representing the same strain are included in some cases. Publicly available read data representing 139 group II isolates (20) were assembled with UGAP as described above, except the assemblies were not screened for contamination or coverage.

### Differentiation of isolates with *rpoB* and core genome SNP phylogenies.

Genome assemblies of isolates within BoNT-producing species were annotated with Prokka (63), and near-full-length (>3,500 nucleotides) *rpoB* genes were parsed from Prokka output files and aligned with MUSCLE (64). A phylogeny was generated with FastTree2 (65) using default parameters and 1,000 bootstrap replicates. The phylogeny was viewed and rooted with an *rpoB* gene extracted from a nonclostridial genome (Escherichia coli J53; GCF_000258865) in FigTree v1.4.2 (http://tree.bio.ed.ac.uk/software/figtree/). If no near-full-length *rpoB* gene was identified in an assembly (*n* = 20), the isolate was assigned to group I, C. sporogenes, or group II on the basis of a literature review and the assignment confirmed using core genome SNP phylogenies. Also, 139 group II isolates published by Weedmark et al. (20) and group III isolate 1873 were not included in the *rpoB* gene analysis.

Core genome single nucleotide polymorphisms (SNPs) were called from BWA-MEM (66) alignments using the UnifiedGenotyper method in GATK (67, 68) in conjunction with NASP (69). Sequencing reads were first aligned to reference genomes (for group I and C. sporogenes, GCF_000022765.1 [Kyoto-F]; for group II, GCF_000020285.1 [Alaska E43]). If sequence reads were not available for a genome, reads were simulated from publicly available genome assemblies with ART (MountRainier) (70) using the following command: art_illumina -ss MSv3 -sam -l 250 -f 75 -m 300 -s 30 -i input -o output. Positions were removed from the analysis if the depth of coverage was less than 10 or if the allele proportion was less than 0.9. Additionally, duplicated regions of the reference genome were identified with NUCmer (71, 72) and removed from the analysis. Maximum likelihood phylogenies were generated from the resulting SNP matrices (bestsnps) with IQ-TREE (v1.4.4) (73) using the ultrafast bootstrap method (74) and the following models: for group I and C. sporogenes, GTR+ASC+G4; for group II, TVM+ASC+G4. Trees were viewed and rooted in FigTree. The consistency index and retention index values were calculated with Phangorn (75).

### Marker identification and multiplex PCR assay development.

Coding region sequences unique to targeted genomes were identified with LS-BSR (76) (Table 2). For the initial LS-BSR analyses, coding regions were predicted with Prodigal (77), clustered with cd-hit (78) (identity threshold of 0.9), and aligned to each genome using the BLAT (79) alignment option. Coding region sequences were considered unique to a targeted species/subgroup if BSR values were greater than 0.8 within the targeted genomes and less than 0.4 outside the targeted genomes. Output from LS-BSR was imported into iTOL (80) to generate heat maps and to pair the data with core genome SNP phylogenies. Primers targeting markers (and excluding nontarget genomes) were designed with Primer3 (81). Multiplex PCRs included primers for the identified markers as well as primers targeting the 16S rRNA gene (82) used as an internal amplification control (Table 3). All PCRs were performed with Promega (Madison, WI, USA) PCR master mix (catalog no. 9PIM750). The PCR program included an initial denaturation step of 2 min at 95°C, followed by 35 cycles of 95°C for 45 s, 57°C for 1 min, and 72°C for 1 min. The PCR program ended with a final extension step of 5 min at 72°C. Final primer concentrations are included in Table 3. PCR products were visualized with gel electrophoresis on 2% agarose gels.

**TABLE 2:** Marker genes and BSR value summary

Locus tag	Annotation	Average BSR value^*a*^					
		C. botulinum group I (*n* = 106)	C. sporogenes (*n* = 27)	Group II E (*n* = 153)	Group II BEF (*n* = 15)	Groups III–VI^*b*^ (*n* = 55)	Other clostridia (*n* = 102)
T259_1133	Hypothetical protein	**0.97**	0.00	0.00	0.00	0.00	0.00
T258_3337	Transglutaminase-like superfamily protein	0.03	**0.98**	0.00	0.00	0.00	0.00
CLH_2632	ATP phosphoribosyltransferase regulatory subunit	0.00	0.01	**0.99**	0.00	0.01	0.01
CLL_A2124	Alpha amylase family protein	0.00	0.00	0.00	**0.96**	0.00	0.00

a BSR values are a measure of the relatedness of a given coding region sequence between two genomes. Boldface values indicate average BSR values for the targeted genomes of each marker. LS-BSR was run with the BLAT alignment option.

b III, C. botulinum group III; IV, C. argentinense; V, C. baratii; VI, C. butyricum.

**TABLE 3:** Multiplex PCR assay information

Locus tag	Target	Forward primer (5′–3′)	Reverse primer (5′–3′)	Final concn in PCR (uM)	Approximate amplicon size (bp)
T259_1133	C. botulinum group I	TGGTGCGATGACAGTTCCATTT	TCATTGGTCCAGATGCAACTCC	0.75	775
T258_3337	C. sporogenes	ATTGGAGTGGACATGCCTGGA	CCCTTTAACCACCGCTTGTTGT	0.5	310
CLH_2632	Group II E	AGGGACGCGGGATCTTGTTTTA	TCCCATCACTCCCCACTAACTCA	0.4	375
CLL_A2124	Group II BEF	GCTTGGACAACATTCAGTGAGGA	GAATGCCTTCTGTTGGCCTCAT	1.5	480
	16S rRNA gene^*a*^	CCAGACTCCTACGGGAGGCAG	CGTATTACCGCGGCTGCTG	0.05	180

a The 16S rRNA gene primers are from Chakravorty et al. (82).

### Isolate and enrichment culture screening.

DNA representing 40 clostridium genomes and E. coli J53 (see Table S1) were subjected to the multiplex PCR assay described above. Additionally, DNA extracted from two enrichment cultures of domesticated-canine fecal samples (40) were screened with the multiplex PCR assay to determine the ability of the assay to identify members of the targeted species/subgroups that may be present in mixed cultures. The assay was considered positive for a member of a targeted species or subgroup if an appropriately sized band was visible on an agarose gel.

### Sensitivity of marker multiplex PCR for a complex sample.

The 16S rRNA gene copy number in DNA extracted (DNeasy kit; Qiagen, Valencia, CA, USA) from a C. botulinum group I isolate (SAMN06022737; BoNT/B SU0305) was estimated with a qPCR assay (83). DNA from this C. botulinum isolate was serially diluted and mixed with DNA extracted from a domesticated-canine fecal sample (40). The concentration of the C. botulinum group I isolate DNA at which the marker multiplex PCR failed to identify the presence of the isolate DNA in the complex mixture was estimated. Absence of a visible, appropriately sized band on a 2% agarose gel (with 4 μl of PCR product loaded) was interpreted as assay failure. The average 16S rRNA gene copy number for C. botulinum genomes was determined with rrnDB (version 4.4.4) (84). The value of nine 16S rRNA copies (median value for nine finished group I genomes available in the database) was used for estimating genome equivalents.

### *ntnh* gene primer development and PCR.

Primers targeting the nontoxic nonhemagglutinin gene (*ntnh*) were developed with PrimerProspector (85) and by visual inspection of *ntnh* gene alignments produced with MUSCLE (64). Primer coverage was evaluated with PrimerProspector by querying the newly designed primers (forward primer, 5′-GAAARRGAWRANTTYTTRCAAGC-3′; reverse primer, 5′-TAYTATRTTYGHTCCTGGDCCAA-3′) against near-full-length (>3,300 bp) *ntnh* genes extracted from genome assemblies. PCRs were performed with Promega PCR master mix under the following cycle conditions: an initial denaturation step of 2 min at 95°C, followed by 35 cycles of 95°C for 30 s, 52°C for 1 min, and 72°C for 1 min. The PCR program ended with a final extension step of 5 min at 72°C. The final primer concentration was 3.0 μM. PCR products were visualized on a 2% agarose gel, and reactions were considered positive if an appropriately sized band was visible on the gel. The amplicon produced from Biosample SAMN03779976 (BoNT/A SU0998) was sequenced to confirm that the primers targeted the appropriate gene. The PCR product was purified using the ExoSAP-IT PCR product cleanup protocol (Affymetrix, Santa Clara, CA, USA), and sequencing reactions were performed using a BigDye Terminator v3.1 kit according to the recommended protocol (Applied Biosystems, Foster City, CA, USA). Products were sequenced on an ABI3130 genetic analyzer (Applied Biosystems, Foster City, CA, USA). Sanger reads were called with PHRED (86, 87) and assembled with PHRAP (www.phrap.org) via CodonCode Aligner (CodonCode Corporation, Centerville, MA, USA). The amplicon sequence was identified with BLAST (61). The amplicon sequence was also aligned to reference sequences with MUSCLE. The resulting alignment was viewed with Jalview (88) and edited to produce a figure with Inkscape (https://inkscape.org).

### Evaluation of published markers.

Markers identified by Dahlsten et al. (37) and Weigand et al. (12) were screened against genome assemblies with LS-BSR (76) to evaluate performance.

### Accession number(s).

Whole-genome sequence data generated as part of this study were submitted to NCBI under BioProject accession number PRJNA353866 (accession numbers of samples are included in Table 1).

### Data availability.

SNP data, *rpoB* gene alignments, and phylogenies are available at https://github.com/chawillia/clade_markers_2017.

## Supplementary Material

Supplemental material
