# Supplementary material for: Differentiating Botulinum Neurotoxin-Producing Clostridia with a Simple, Multiplex PCR Assay
Source: Appl Environ Microbiol. 2017 Aug 31;83(18):e00806-17. doi: 10.1128/AEM.00806-17 (PMC5583490; doi:10.1128/AEM.00806-17)
Supplement: Supplemental material [file supp_83_18_e00806-17__index.html]

Supplemental material 

# Differentiating Botulinum Neurotoxin-Producing Clostridia with a Simple, Multiplex PCR Assay

## Supplemental material

- Supplemental file 1 -

  Assessment of multiplex marker PCR amplicons with gel electrophoresis (Fig. S1), estimation of the sensitivity of the marker multiplex PCR assay for a complex DNA sample (Fig. S2), MUSCLE alignment of *ntnh* gene sequences (Fig. S3), assessment of *ntnh* gene PCR assay with gel electrophoresis (Fig. S4), summary of PCR assay results (Table S1), and *in silico* evaluation of *ntnh* primers (Table S2).

  PDF, 5.3M
- Supplemental file 2 -

  BioSample details (Data Set S1) and genome details (Data Set S2).

  XLSX, 278K
