# Supplementary material for: Differentiating Botulinum Neurotoxin-Producing Clostridia with a Simple, Multiplex PCR Assay
Source: Appl Environ Microbiol. 2017 Aug 31;83(18):e00806-17. doi: 10.1128/AEM.00806-17 (PMC5583490; doi:10.1128/AEM.00806-17)
Supplement: Supplemental material [file AEM.00806-17_zam018178058s1.pdf]

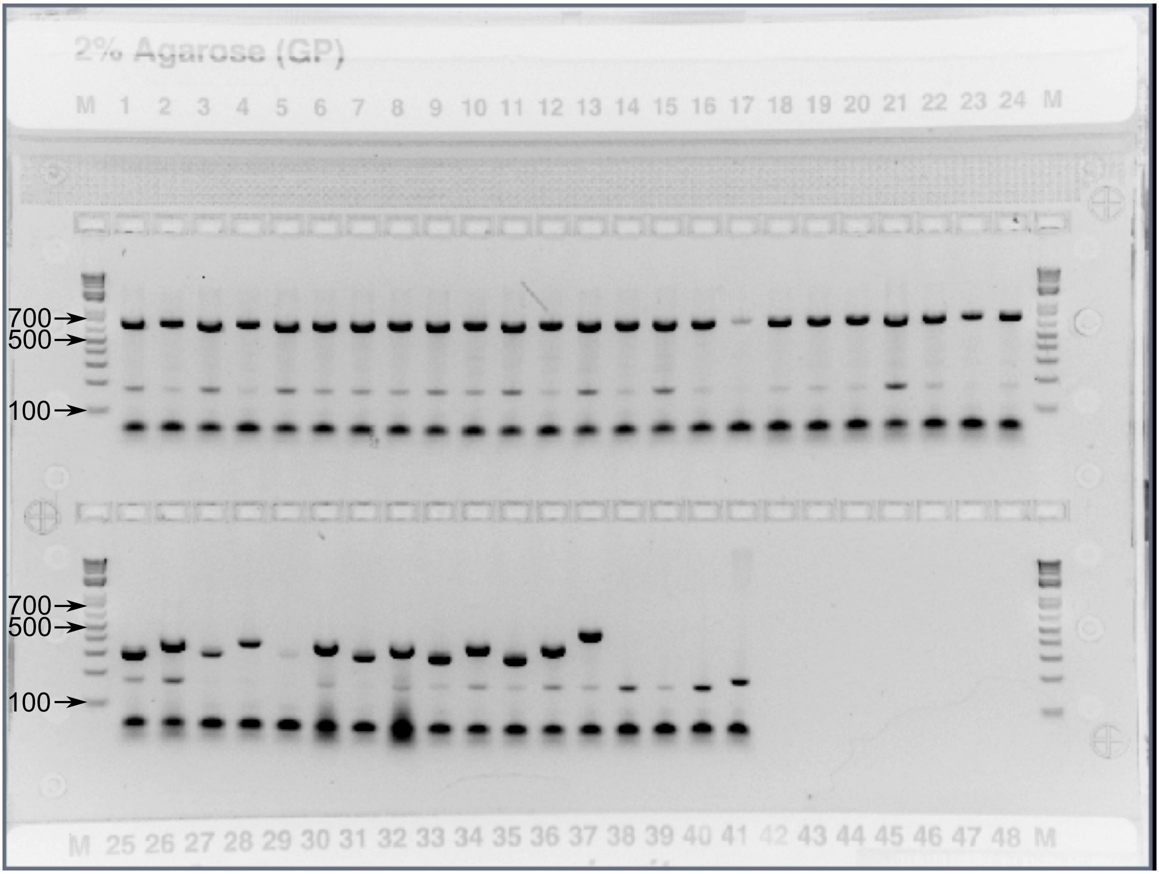

Lane	Sample	Species	Lane	Sample	Species
1	SU0998	*C. botulinum* Group I	22	Man216	*C. botulinum* Group I
2	okra	*C. botulinum* Group I	23	CDC 1656	*C. botulinum* Group I
3	SU1274	*C. botulinum* Group I	24	AM1295	*C. botulinum* Group I
4	ATCC 25763	*C. botulinum* Group I	25	ATCC_51387	*C. sporogenes*
5	SU1887	*C. botulinum* Group I	26	Prevot Ped 4	*C. botulinum* Group II "E"
6	Prevot 1542	*C. botulinum* Group I	27	Prevot 594	*C. sporogenes*
7	SU1937	*C. botulinum* Group I	28	L-572	*C. botulinum* Group II "E"
8	CDC795	*C. botulinum* Group I	29	Prevot 1662	*C. sporogenes*
9	SU1306	*C. botulinum* Group I	30	Bac-01-03998	*C. botulinum* Group II "E"
10	SU0635W	*C. botulinum* Group I	31	AM553	*C. sporogenes*
11	SU1169	*C. botulinum* Group I	32	Bac-02-06430	*C. botulinum* Group II "E"
12	SU0632	*C. botulinum* Group I	33	AM1195	*C. sporogenes*
13	SU1033	*C. botulinum* Group I	34	Bac-03-06093	*C. botulinum* Group II "E"
14	SU1297	*C. botulinum* Group I	35	AM370	*C. sporogenes*
15	SU1575NT	*C. botulinum* Group I	36	Bac-04-16057	*C. botulinum* Group II "E"
16	SU0972	*C. botulinum* Group I	37	Eklund 17B	*C. botulinum* Group II "BEF"
17	SU0729	*C. botulinum* Group I	38	1873	*C. botulinum* Group III
18	BrDuraAf	*C. botulinum* Group I	39	BL5262	*C. butyricum*
19	CDC1744	*C. botulinum* Group I	40	SU1074NT	*P. bifermentans*
20	SU0305	*C. botulinum* Group I	41	J53	*E. coli*
21	Loch Maree	*C. botulinum* Group I			

Fig. S1. Assessment of multiplex marker PCR amplicons with gel electrophoresis. DNA extractions representing 41 bacterial isolates were tested with the multiplex marker PCR assay. Target species/subgroup amplicons have the following approximate sizes: Group I *C. botulinum* – 775 bp, *C. sporogenes* – 310 bp, Group II E subgroup – 375 bp, Group II BEF subgroup – 480 bp, internal amplification control (16S rRNA gene) – 180 bp. Two bands indicate a positive reaction for one of the targeted species/subgroups. The numbers to the right of the gel indicate the size (bp) of the ladder bands. The PCR assay correctly identified all 41 strains.

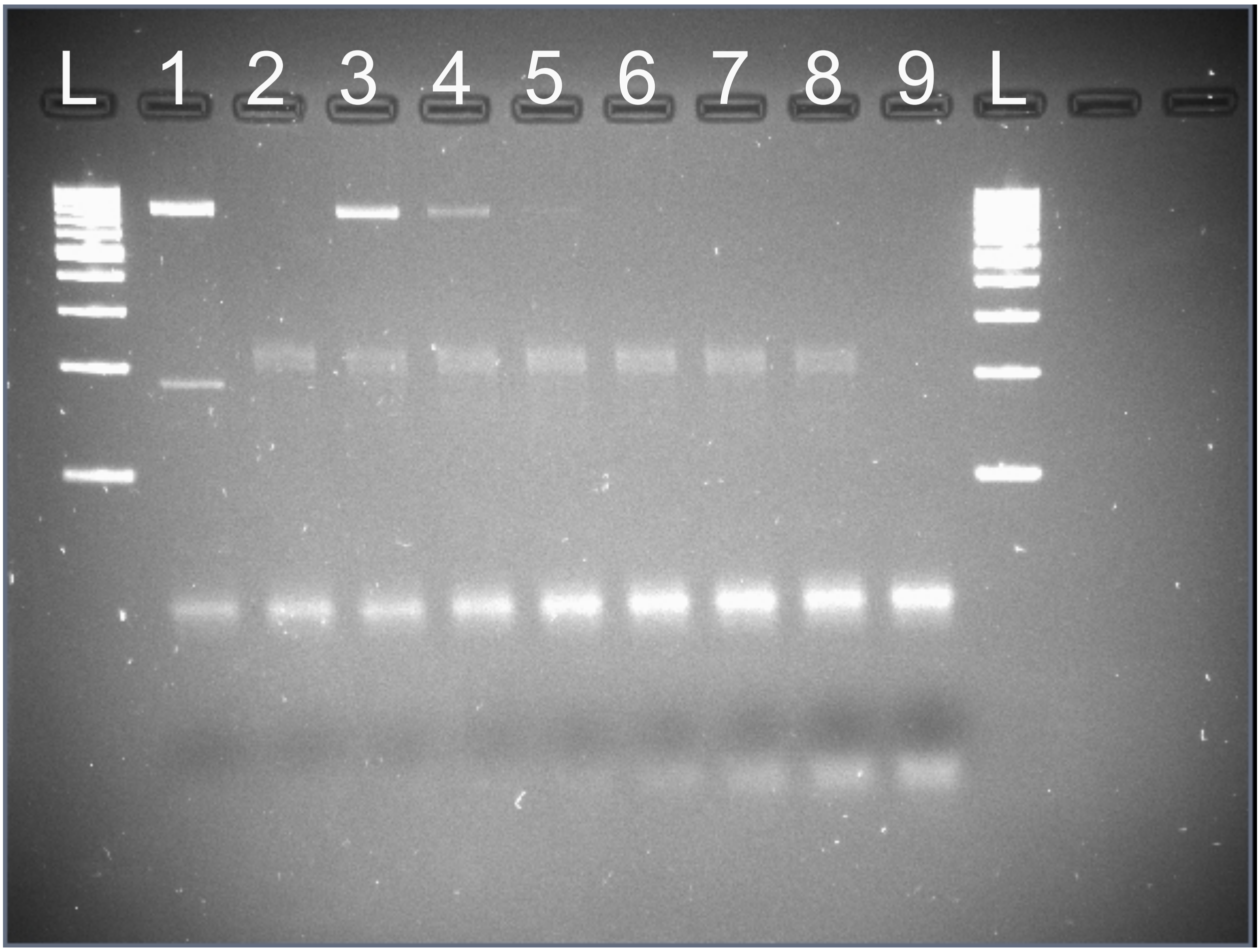

Well	Sample	*C. botulinum* Group I DNA concentration estimate (16S rRNA gene copy number)	*C. botulinum* Group I DNA concentration estimate (genome equivalents)
L	ladder	NA	NA
1	*C. botulinum* Group I - SU0305	1.0E+06	1.1E+05
2	Domesticated canine fecal sample	NA	NA
3	mix of Cbot and canine fecal sample DNA	2.1E+05	2.3E+04
4	mix of Cbot and canine fecal sample DNA	1.7E+04	1.8E+03
5	mix of Cbot and canine fecal sample DNA	2.4E+03	2.6E+02
6	mix of Cbot and canine fecal sample DNA*	2.4E+02	2.6E+01
7	mix of Cbot and canine fecal sample DNA*	2.4E+01	2.6E+00
8	mix of Cbot and canine fecal sample DNA*	2.4E+00	2.6E-01
9	negative control	NA	NA
L	ladder	NA	NA

*concentration estimates based upon serial dilutions

Fig. S2. The sensitivity of the marker multiplex PCR assay for a complex DNA sample was estimated by mixing DNA extracted from a *C. botulinum* Group I isolate (SU0305 - SAMN06022737) into DNA extracted from a canine fecal sample. The *C. botulinum* Group I isolate DNA was serially diluted and quantified with a qPCR assay before mixing with the canine fecal sample DNA. The multiplex PCR assay was run on the resulting DNA mixtures. 4 ul of PCR product was run on a 2% agarose gel. Though a very faint band was produced on the gel, the assay successfully identified the *C. botulinum* Group I isolate DNA when ~2400 16S rRNA gene copies (~270 genome equivalents) were present in the reaction.

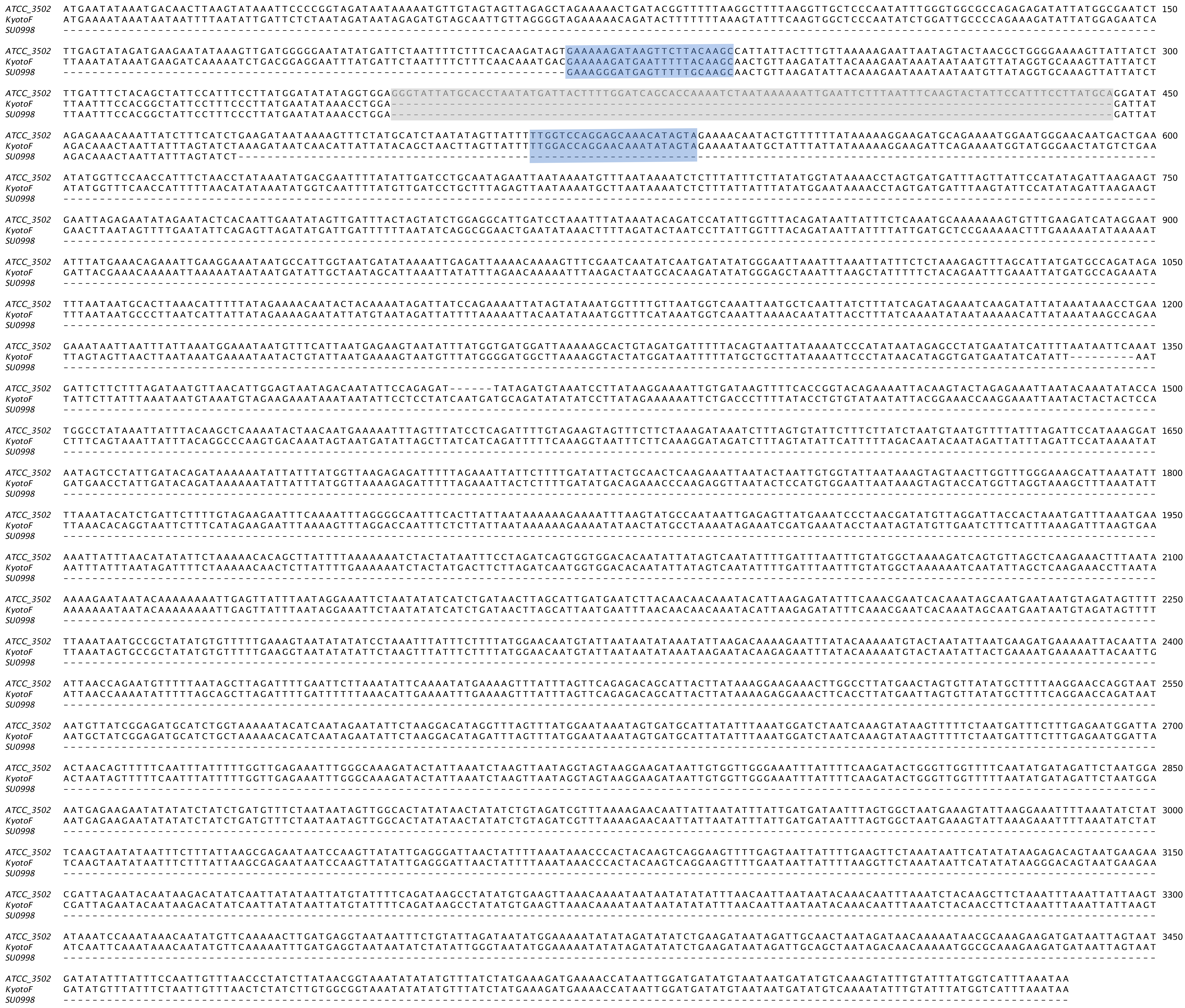

Fig. S3. MUSCLE alignment of *ntnh* gene sequences. The alignment includes *ntnh* gene sequences from the following isolates: ATCC 3502 (*bont*/A1 gene, *ha*+ toxin gene cluster), Kyoto-F (*bont*/A2 gene, *orfX* + toxin gene cluster), SU0998 (*bont*/A2 gene, *orfX*+ toxin gene cluster). The sequence representing SU0998 was generated by sequencing the amplicon produced with the primers designed in this study. The blue shaded regions indicate the primer-targeted regions of the gene. The gray shaded region indicates the portion of the gene encoding the nLoop; this region of the gene is absent in *ntnh* genes associated with *orfX*+ toxin gene clusters.

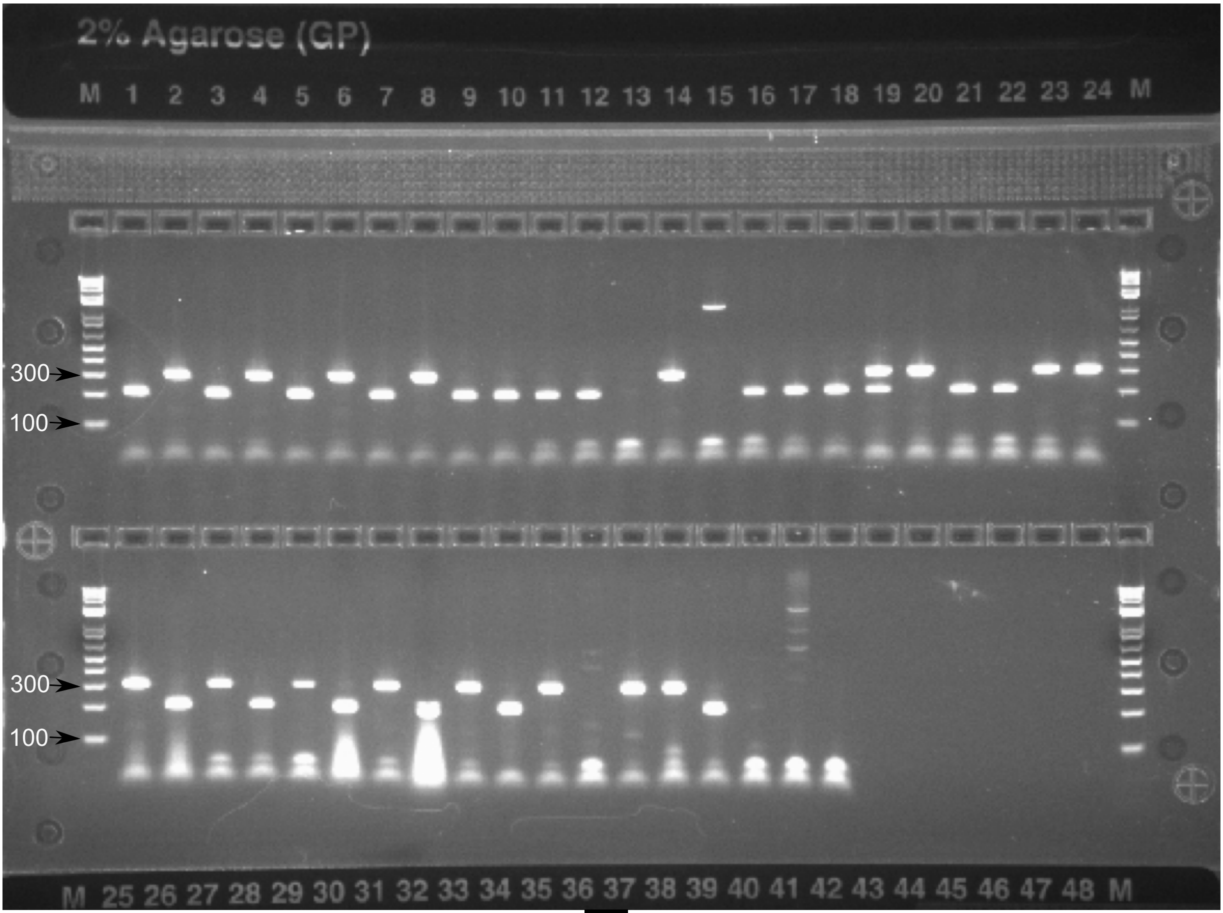

Lane	Sample	BoNT	orfX or ha
1	SU0998	A	orfX
2	okra	B	ha
3	SU1274	A	orfX
4	ATCC 25763	A	ha
5	SU1887	A	orfX
6	Prevot 1542	B	ha
7	SU1937	A	orfX
8	CDC795	B	ha
9	SU1306	Af	orfX
10	SU0635W	A	orfX
11	SU1169	A	orfX
12	SU0632	F	orfX
13	SU1033	NT	
14	SU1297	B	ha
15	SU1575NT	NT	
16	SU0972	A	orfX
17	SU0729	A	orfX
18	BrDuraAf	Af	orfX
19	CDC1744	A(B)	orfX and ha
20	SU0305	B	ha
21	Loch Maree	A	orfX

Lane	Sample	BoNT	orfX or ha
22	Man216	F	orfX
23	CDC 1656	B	ha
24	AM1295	A	ha
25	ATCC_51387	B	ha
26	Prevot Ped 4	E	orfX
27	Prevot 594	B	ha
28	L-572	E	orfX
29	Prevot 1662	B	ha
30	Bac-01-03998	E	orfX
31	AM553	B	ha
32	Bac-02-06430	E	orfX
33	AM1195	B	ha
34	Bac-03-06093	E	orfX
35	AM370	B	ha
36	Bac-04-16057	NT	
37	Eklund 17B	B	ha
38	1873	D	ha
39	BL5262	E	orfX
40	SU1074NT	NT	
41	J53	NT	
42	negative control	NT	

Fig. S4. Assessment of *ntnh* gene PCR assay with gel electrophoresis. DNA extractions representing 41 bacterial isolates were tested with the *ntnh* gene PCR assay. An appropriately-sized band was present for all isolates in which a *bont* gene (and, thus, an *ntnh* gene) was found in the genome assembly. Two different sized bands are present. The approximately 325 bp band represents *ntnh* genes associated with *ha*+ toxin gene clusters; while the approximately 225 bp band represents *ntnh* genes associated with *orfX*+ toxin gene clusters. Lane 15 includes a non-toxic *C. botulinum* Group I strain, and the large amplicon is not the appropriate size for the *ntnh* gene PCR assay and is not indicative of the presence of an *ntnh* gene. Lane 19 includes a BoNT/A1(B) strain (A1-*orfX*+, (B)-*ha*+), which produces two bands on the gel. Both *orfX*+ (lane 17) and ha+ (lane 24) BoNT/A1 strains are included in the analysis.

TABLE S1 Summary of PCR assay results

				PCR target and result				
**Biosample**	**Infraspecific Name**	**Species/Subgroup**	**BoNT**	**T259_1133**	**T258_3337**	**CLH_2632**	**CLL_A2124**	***ntnh* gene**
SAMN03779976	SU0998	*C. botulinum* Group I	A	positive	negative	negative	negative	positive
SAMN03779963	SU1274	*C. botulinum* Group I	A	positive	negative	negative	negative	positive
SAMN03779965	SU1887	*C. botulinum* Group I	A	positive	negative	negative	negative	positive
SAMN03779962	SU1937	*C. botulinum* Group I	A	positive	negative	negative	negative	positive
SAMN03779981	SU1306	*C. botulinum* Group I	Af	positive	negative	negative	negative	positive
SAMN03779983	SU1169	*C. botulinum* Group I	A	positive	negative	negative	negative	positive
SAMN06022749	SU1033	*C. botulinum* Group I	NT	positive	negative	negative	negative	negative
SAMN03780006	SU1575NT	*C. botulinum* Group I	NT	positive	negative	negative	negative	negative
SAMN03779955	SU0279	*C. botulinum* Group I	A	positive	negative	negative	negative	positive
SAMN03779956	CDC1744	*C. botulinum* Group I	A(B)	positive	negative	negative	negative	positive
SAMN02603536	Loch Maree	*C. botulinum* Group I	A	positive	negative	negative	negative	positive
SAMN06022746	CDC 1656	*C. botulinum* Group I	B	positive	negative	negative	negative	positive
SAMN02603535	okra	*C. botulinum* Group I	B	positive	negative	negative	negative	positive
SAMN03862115	ATCC 25763	*C. botulinum* Group I	A	positive	negative	negative	negative	positive
SAMN06022747	Prevot 1542	*C. botulinum* Group I	B	positive	negative	negative	negative	positive
SAMN03779987	CDC795	*C. botulinum* Group I	B	positive	negative	negative	negative	positive
SAMN03779980	SU0635W	*C. botulinum* Group I	A	positive	negative	negative	negative	positive
SAMN06022739	SU0632	*C. botulinum* Group I	F	positive	negative	negative	negative	positive
SAMN06022738	SU1297	*C. botulinum* Group I	B	positive	negative	negative	negative	positive
SAMN06022736	SU0972	*C. botulinum* Group I	A	positive	negative	negative	negative	positive
SAMN06022741	BrDuraAf	*C. botulinum* Group I	Af	positive	negative	negative	negative	positive
SAMN06022737	SU0305	*C. botulinum* Group I	B	positive	negative	negative	negative	positive
SAMN06022740	Man216	*C. botulinum* Group I	F	positive	negative	negative	negative	positive
SAMN03779954	AM1295	*C. botulinum* Group I	A	positive	negative	negative	negative	positive
SAMN03169465	ATCC_51387	*C. sporogenes*	B	negative	positive	negative	negative	positive
SAMN03222821	Prevot 594	*C. sporogenes*	B	negative	positive	negative	negative	positive
SAMN03169475	Prevot 1662	*C. sporogenes*	B	negative	positive	negative	negative	positive
SAMN03779991	AM553	*C. sporogenes*	B	negative	positive	negative	negative	positive
SAMN03779989	AM1195	*C. sporogenes*	B	negative	positive	negative	negative	positive
SAMN03779990	AM370	*C. sporogenes*	B	negative	positive	negative	negative	positive
SAMN06022748	Prevot Ped 4	*C. botulinum* Group II "E"	E	negative	negative	positive	negative	positive
SAMN03779994	L-572	*C. botulinum* Group II "E"	E	negative	negative	positive	negative	positive
SAMN06022742	Bac-01-03998	*C. botulinum* Group II "E"	E	negative	negative	positive	negative	positive
SAMN06022743	Bac-02-06430	*C. botulinum* Group II "E"	E	negative	negative	positive	negative	positive
SAMN06022744	Bac-03-06093	*C. botulinum* Group II "E"	E	negative	negative	positive	negative	positive
SAMN06022745	Bac-04-16057	*C. botulinum* Group II "E"	NT	negative	negative	positive	negative	negative
SAMN02603538,SAMEA2272780,SAMN03731052	Eklund 17B	*C. botulinum* Group II "BEF"	B	negative	negative	negative	positive	positive
SAMN02470277	1873	*C. botulinum* Group III	D	negative	negative	negative	negative	positive
SAMN02470281	BL5262	*C. butyricum* Group VI	E	negative	negative	negative	negative	positive
SAMN06022750	SU1074NT	NA	NT	negative	negative	negative	negative	negative
SAMN02470906	J53	*E. coli*	NT	negative	negative	negative	negative	negative

TABLE S2 ***In silico*** **evaluation of** ***ntnh*** **primers**

Biosample	Organism Name	Infraspecific Name	Species/Subgroup	BoNT type	*orfX* or *ha*	Approximate Amplicon Size
SAMN06022736	Clostridium botulinum	SU0972	*C. botulinum* Group I	A	*orfX*	225
SAMN02603536	Clostridium botulinum A3 str. Loch Maree	Loch Maree	*C. botulinum* Group I	A	*orfX*	225
SAMN02603539	Clostridium botulinum Ba4 str. 657	657	*C. botulinum* Group I	A	*orfX*	225
SAMN02603540	Clostridium botulinum A2 str. Kyoto	Kyoto	*C. botulinum* Group I	A	*orfX*	225
SAMN02436237	Clostridium botulinum NCTC 2916	NCTC 2916	*C. botulinum* Group I	A	*orfX*	225
SAMN02470269	Clostridium botulinum Af84	Af84	*C. botulinum* Group I	A	*orfX*	225
SAMN02869849	Clostridium botulinum A2B3 87	A2B3 87	*C. botulinum* Group I	A	*orfX*	225
SAMN03222820	Clostridium botulinum CDC_1436	CDC_1436	*C. botulinum* Group I	A	*orfX*	225
SAMN03779961	Clostridium botulinum	Mauritius	*C. botulinum* Group I	A	*orfX*	225
SAMN03779962	Clostridium botulinum	SU1937	*C. botulinum* Group I	A	*orfX*	225
SAMN03779963	Clostridium botulinum	SU1274	*C. botulinum* Group I	A	*orfX*	225
SAMN03779964	Clostridium botulinum	SU1917	*C. botulinum* Group I	A	*orfX*	225
SAMN03779965	Clostridium botulinum	SU1887	*C. botulinum* Group I	A	*orfX*	225
SAMN03779966	Clostridium botulinum	SU1275	*C. botulinum* Group I	A	*orfX*	225
SAMN03779967	Clostridium botulinum	SU1934	*C. botulinum* Group I	A	*orfX*	225
SAMN03779968	Clostridium botulinum	SU1259	*C. botulinum* Group I	A	*orfX*	225
SAMN03779969	Clostridium botulinum	SU1891	*C. botulinum* Group I	A	*orfX*	225
SAMN03779970	Clostridium botulinum	SU1054	*C. botulinum* Group I	A	*orfX*	225
SAMN03779971	Clostridium botulinum	SU1072	*C. botulinum* Group I	A	*orfX*	225
SAMN03779972	Clostridium botulinum	SU1064	*C. botulinum* Group I	A	*orfX*	225
SAMN03779983	Clostridium botulinum	SU1169	*C. botulinum* Group I	A	*orfX*	225
SAMN03779973	Clostridium botulinum	SU1074	*C. botulinum* Group I	A	*orfX*	225
SAMN03779974	Clostridium botulinum	SU1112	*C. botulinum* Group I	A	*orfX*	225
SAMN03779984	Clostridium botulinum	SU0945	*C. botulinum* Group I	A	*orfX*	225
SAMN03779975	Clostridium botulinum	SU0801	*C. botulinum* Group I	A	*orfX*	225
SAMN03779977	Clostridium botulinum	SU0807	*C. botulinum* Group I	A	*orfX*	225
SAMN03779976	Clostridium botulinum	SU0998	*C. botulinum* Group I	A	*orfX*	225
SAMN03779978	Clostridium botulinum	SU0994	*C. botulinum* Group I	A	*orfX*	225
SAMN03779979	Clostridium botulinum	SU0634	*C. botulinum* Group I	A	*orfX*	225
SAMN03779955	Clostridium botulinum	SU0729	*C. botulinum* Group I	A	*orfX*	225
SAMN03779980	Clostridium botulinum	SU0635W	*C. botulinum* Group I	A	*orfX*	225
SAMN02952937	Clostridium botulinum CDC66177	CDC66177	*C. botulinum* Group II "BEF"	E	*orfX*	225
SAMN06022742	Clostridium botulinum	Bac-01-03998	*C. botulinum* Group II "E"	E	*orfX*	225
SAMN06022743	Clostridium botulinum	Bac-02-06430	*C. botulinum* Group II "E"	E	*orfX*	225
SAMN06022748	Clostridium botulinum	Prevot Ped 4	*C. botulinum* Group II "E"	E	*orfX*	225
SAMN02603537	Clostridium botulinum E3 str. Alaska E43	Alaska E43	*C. botulinum* Group II "E"	E	*orfX*	225
SAMN02470262	Clostridium botulinum E1 str. 'BoNT E Beluga'	BoNT E Beluga	*C. botulinum* Group II "E"	E	*orfX*	225
SAMN02471792	Clostridium botulinum CB11/1-1	CB11/1-1	*C. botulinum* Group II "E"	E	*orfX*	225
SAMN03284354	Clostridium botulinum	NCTC 8266	*C. botulinum* Group II "E"	E	*orfX*	225
SAMN03284339	Clostridium botulinum	NCTC 8550	*C. botulinum* Group II "E"	E	*orfX*	225
SAMN03283906	Clostridium botulinum	NCTC 11219	*C. botulinum* Group II "E"	E	*orfX*	225
SAMN03787461	Clostridium botulinum	ATCC 9564	*C. botulinum* Group II "E"	E	*orfX*	225
SAMN03862114	Clostridium botulinum	ATCC 17786	*C. botulinum* Group II "E"	E	*orfX*	225
SAMN03779999	Clostridium botulinum	211	*C. botulinum* Group II "E"	E	*orfX*	225

SAMN03779993	Clostridium botulinum	CDC KA-95B	*C. botulinum* Group II "E"	E	*orfX*	225
SAMN03779994	Clostridium botulinum	L-572	*C. botulinum* Group II "E"	E	*orfX*	225
SAMN03779998	Clostridium botulinum	CDC 5247	*C. botulinum* Group II "E"	E	*orfX*	225
SAMN03779995	Clostridium botulinum	Prevot Ped 1	*C. botulinum* Group II "E"	E	*orfX*	225
SAMN03780000	Clostridium botulinum	Prevot R81-3A	*C. botulinum* Group II "E"	E	*orfX*	225
SAMN03779996	Clostridium botulinum	ATCC 9564	*C. botulinum* Group II "E"	E	*orfX*	225
SAMN03779997	Clostridium botulinum	K15	*C. botulinum* Group II "E"	E	*orfX*	225
SAMN03780001	Clostridium botulinum	K3	*C. botulinum* Group II "E"	E	*orfX*	225
SAMN02436238	Clostridium butyricum 5521	5521	*C. butyricum* Group VI	E	*orfX*	225
SAMN02470281	Clostridium butyricum E4 str. BoNT E BL5262	BoNT E BL5262	C. butyricum Group VI	E	*orfX*	225
SAMN06022739	Clostridium botulinum	SU0632	*C. botulinum* Group I	F	*orfX*	225
SAMN06022740	Clostridium botulinum	Man216	*C. botulinum* Group I	F	*orfX*	225
SAMN02603533	Clostridium botulinum F str. Langeland	Langeland	*C. botulinum* Group I	F	*orfX*	225
SAMN02603020	Clostridium botulinum F str. 230613	230613	*C. botulinum* Group I	F	*orfX*	225
SAMN02436292	Clostridium botulinum Bf	Bf	*C. botulinum* Group I	F	*orfX*	225
SAMN02470269	Clostridium botulinum Af84	Af84	*C. botulinum* Group I	F	*orfX*	225
SAMN02470269	Clostridium botulinum Af84	Af84	*C. botulinum* Group I	F	*orfX*	225
SAMN02951883	Clostridium botulinum CDC54088	CDC54088	*C. botulinum* Group I	F	*orfX*	225
SAMN02951881	Clostridium botulinum CDC54085	CDC54085	*C. botulinum* Group I	F	*orfX*	225
SAMN03418622	Clostridium botulinum	Walls 8G	*C. botulinum* Group I	F	*orfX*	225
SAMN03779988	Clostridium botulinum	An436	*C. botulinum* Group I	F	*orfX*	225
SAMN03779992	Clostridium botulinum	Walls 8G	*C. botulinum* Group I	F	*orfX*	225
SAMN03222822	Clostridium botulinum 202F	202F	*C. botulinum* Group II "BEF"	F	*orfX*	225
SAMN03787460	Clostridium botulinum	ATCC 23387	*C. botulinum* Group II "BEF"	F	*orfX*	225
SAMN03114998	Clostridium botulinum	CFSAN024410	*C. botulinum* Group I	FA	*orfX*	225
SAMN06022744	Clostridium botulinum	Bac-03-06093	*C. botulinum* Group II "E"	E	*orfX*	225
SAMN02436237	Clostridium botulinum NCTC 2916	NCTC 2916	*C. botulinum* Group I	(B)	*ha*	325
SAMN01816399	Clostridium botulinum CFSAN001628	CFSAN001628	*C. botulinum* Group I	(B)	*ha*	325
SAMN02603532	Clostridium botulinum A str. ATCC 19397	ATCC 19397	*C. botulinum* Group I	A	*ha*	325
SAMN02603534	Clostridium botulinum A str. Hall	Hall	*C. botulinum* Group I	A	*ha*	325
SAMEA1705919	Clostridium botulinum A str. ATCC 3502	ATCC 3502	*C. botulinum* Group I	A	*ha*	325
SAMN03787462	Clostridium botulinum	ATCC 17862	*C. botulinum* Group I	A	*ha*	325
SAMN03862115	Clostridium botulinum	ATCC 25763	*C. botulinum* Group I	A	*ha*	325
SAMN03779946	Clostridium botulinum	VPI 7124	*C. botulinum* Group I	A	*ha*	325
SAMN03779947	Clostridium botulinum	ATCC 449	*C. botulinum* Group I	A	*ha*	325
SAMN03779948	Clostridium botulinum	KF Meyer 126	*C. botulinum* Group I	A	*ha*	325
SAMN03779949	Clostridium botulinum	Prevot 910	*C. botulinum* Group I	A	*ha*	325
SAMN03779950	Clostridium botulinum	Prevot Dewping	*C. botulinum* Group I	A	*ha*	325
SAMN03779951	Clostridium botulinum	Prevot 697B	*C. botulinum* Group I	A	*ha*	325
SAMN03779952	Clostridium botulinum	McClung 844	*C. botulinum* Group I	A	*ha*	325
SAMN03779953	Clostridium botulinum	KF Meyer 33	*C. botulinum* Group I	A	*ha*	325
SAMN03779954	Clostridium botulinum	AM1295	*C. botulinum* Group I	A	*ha*	325
SAMEA2272270	Clostridium botulinum H04402 065	H04402 065	*C. botulinum* Group I	A	*ha*	325
SAMD00036773	Clostridium botulinum B str. Osaka05	Osaka05	*C. sporogenes*	B	*ha*	325
SAMN03222821	Clostridium botulinum Prevot_594	Prevot_594	*C. sporogenes*	B	*ha*	325
SAMN02854325	Clostridium botulinum B2 450	B2 450	*C. sporogenes*	B	*ha*	325
SAMN03779989	Clostridium botulinum	AM1195	*C. sporogenes*	B	*ha*	325

SAMN03779990	Clostridium botulinum	AM370	*C. sporogenes*	B	*ha*		325
SAMN03779991	Clostridium botulinum	AM553	*C. sporogenes*	B	*ha*		325
SAMN06022737	Clostridium botulinum	SU0305	*C. botulinum* Group I	B	*ha*		325
SAMN06022738	Clostridium botulinum	SU1297	*C. botulinum* Group I	B	*ha*		325
SAMN06022746	Clostridium botulinum	CDC 1656	*C. botulinum* Group I	B	*ha*		325
SAMN06022747	Clostridium botulinum	Prevot 1542	*C. botulinum* Group I	B	*ha*		325
SAMN02603535	Clostridium botulinum B1 str. Okra	Okra	*C. botulinum* Group I	B	*ha*		325
SAMN02603539	Clostridium botulinum Ba4 str. 657	657	*C. botulinum* Group I	B	*ha*		325
SAMN02436292	Clostridium botulinum Bf	Bf	*C. botulinum* Group I	B	*ha*		325
SAMN02869849	Clostridium botulinum A2B3 87	A2B3 87	*C. botulinum* Group I	B	*ha*		325
SAMN03114998	Clostridium botulinum	CFSAN024410	*C. botulinum* Group I	B	*ha*		325
SAMN03222820	Clostridium botulinum CDC_1436	CDC_1436	*C. botulinum* Group I	B	*ha*		325
SAMN03274242	Clostridium botulinum	277-00	*C. botulinum* Group I	B	*ha*		325
SAMN03787459	Clostridium botulinum	ATCC 17843	*C. botulinum* Group I	B	*ha*		325
SAMN03787463	Clostridium botulinum	ATCC 7949	*C. botulinum* Group I	B	*ha*		325
SAMN03779986	Clostridium botulinum	ATCC 17843 (B5)	*C. botulinum* Group I	B	*ha*		325
SAMN03779988	Clostridium botulinum	An436	*C. botulinum* Group I	B	*ha*		325
SAMN03779985	Clostridium botulinum	Hall 80	*C. botulinum* Group I	B	*ha*		325
SAMN03779987	Clostridium botulinum	CDC 795	*C. botulinum* Group I	B	*ha*		325
SAMN02603538	Clostridium botulinum B str. Eklund 17B (NRP)	Eklund 17B	*C. botulinum* Group II "BEF"	B	*ha*		325
SAMEA2272780	Clostridium botulinum B str. Eklund 17B (NRP)	B str. Eklund 17B(NRP)	*C. botulinum* Group II "BEF"	B	*ha*		325
SAMN03012885	Clostridium botulinum	KAPB-3	*C. botulinum* Group II "BEF"	B	*ha*		325
SAMN03012839	Clostridium botulinum	DB-2	*C. botulinum* Group II "BEF"	B	*ha*		325
SAMN02436293	Clostridium botulinum C str. Eklund	Eklund	*C. botulinum* Group III	C	*ha*		325
SAMN02470655	Clostridium botulinum V891	V891	*C. botulinum* Group III	C	*ha*		325
SAMN02603671	Clostridium botulinum BKT015925	BKT015925	*C. botulinum* Group III	C	*ha*		325
SAMN02593749	Clostridium botulinum C/D str. BKT2873	BKT2873	*C. botulinum* Group III	C/D	*ha*		325
SAMN02593748	Clostridium botulinum C/D str. BKT75002	BKT75002	*C. botulinum* Group III	C/D	*ha*		325
SAMN02593746	Clostridium botulinum C/D str. Sp77	Sp77	*C. botulinum* Group III	C/D	*ha*		325
SAMN03876597	Clostridium botulinum	43243-CD	*C. botulinum* Group III	C/D	*ha*		325
SAMN03876592	Clostridium botulinum	12LNRI-CD	*C. botulinum* Group III	C/D	*ha*		325
SAMN03876598	Clostridium botulinum	48212-CD	*C. botulinum* Group III	C/D	*ha*		325
SAMN03876596	Clostridium botulinum	38028-CD	*C. botulinum* Group III	C/D	*ha*		325
SAMN03876593	Clostridium botulinum	12LNR10-CD	*C. botulinum* Group III	C/D	*ha*		325
SAMN03876595	Clostridium botulinum	29401-CD	*C. botulinum* Group III	C/D	*ha*		325
SAMN03876594	Clostridium botulinum	12LNR13-CD	*C. botulinum* Group III	C/D	*ha*		325
SAMN03876601	Clostridium botulinum	55741-CD	*C. botulinum* Group III	C/D	*ha*		325
SAMN03876600	Clostridium botulinum	50867-CD	*C. botulinum* Group III	C/D	*ha*		325
SAMN03876599	Clostridium botulinum	49511-CD	*C. botulinum* Group III	C/D	*ha*		325
SAMN03876605	Clostridium botulinum	71840-CD	*C. botulinum* Group III	C/D	*ha*		325
SAMN03876602	Clostridium botulinum	58272-CD	*C. botulinum* Group III	C/D	*ha*		325
SAMN02593747	Clostridium botulinum D str. 16868	16868	*C. botulinum* Group III	D	*ha*		325
SAMN03876608	Clostridium botulinum	51714-DC	*C. botulinum* Group III	D/C	*ha*		325
SAMN03876607	Clostridium botulinum	47295-DC	*C. botulinum* Group III	D/C	*ha*		325
SAMN02470277	Clostridium botulinum D str. 1873	1873	*C. botulinum* Group III	D	*ha*		325
SAMN03222824	Clostridium argentinense CDC 2741	CDC 2741	*C. argentinense* Group IV	G	*ha*		325
SAMD00024166	Clostridium botulinum	strain=111	*C. botulinum* Group I	unknown	*orfX*	NO AMPLICON	
